# Is cognitive control of perception and action via attentional focus moderated by motor imagery?

**DOI:** 10.1186/s40359-023-01047-z

**Published:** 2023-01-16

**Authors:** Behzad Bazgir, Alireza Shamseddini, Jennifer A. Hogg, Farhad Ghadiri, Moslem Bahmani, Jed A. Diekfuss

**Affiliations:** 1 Exercise Physiology Research Center, Life Style Institute, Baqiyatallah University of Medical Sciences, Tehran, Iran; 2 Department of Motor Behavior, Kharazmi University, Tehran, Iran; 3 Department of Health and Human Performance, The University of Tennessee Chattanooga, Chattanooga, TN USA; 4 Emory Sports Performance And Research Center (SPARC), Flowery Branch, GA USA; 5 Emory Sports Medicine Center, Atlanta, GA USA; 6 Department of Orthopaedics, Emory University School of Medicine, Atlanta, GA USA

**Keywords:** Kinesthetic motor imagery, Visual imagery, Internal focus, External focus, Air-pistol shooting

## Abstract

Motor imagery (MI) has emerged as an individual factor that may modulate the effects of attentional focus on motor skill performance. In this study, we investigated whether global MI, as well as its components (i.e., kinesthetic MI, internal visual MI, and external visual MI) moderate the effect of attentional focus on performance in a group of ninety-two young adult novice air-pistol shooters (age: M = 21.87, SD = 2.54). After completing the movement imagery questionnaire-3 (MIQ-3), participants were asked to complete a pistol shooting experiment in three different attentional focus conditions: (1) No focus instruction condition (control condition with no verbal instruction) (2) an internal focus instruction condition, and (3) an external focus condition. Shot accuracy, performance time, and aiming trace speed (i.e., stability of hold or weapon stability) were measured as the performance variables. Results revealed that shot accuracy was significantly poorer during internal relative to control focus condition. In addition, performance time was significantly higher during external relative to both control and internal condition. However, neither global MI, nor its subscales, moderated the effects of attentional focus on performance. This study supports the importance of attentional focus for perceptual and motor performance, yet global MI and its modalities/perspectives did not moderate pistol shooting performance. This study suggests that perception and action are cognitively controlled by attentional mechanisms, but not motor imagery. Future research with complementary assessment modalities is warranted to extend the present findings.

**Supplementary Information:**

The online version contains supplementary material available at 10.1186/s40359-023-01047-z.

## Background

Individuals perform motor tasks with differing levels of attention. Traditional views considered attention as a passive spotlight or gatekeeper, acting prior to perceptual processing to merely filter out undesirable inputs in favor of others (i.e., it does not affect cognitive control of perception) [1–6]. In contrast, attention currently is viewed as a dynamic mechanism that actively modulates cognitive control of perceptual computations in almost all stages or levels of processing [7–10], see also [11–14]. At the neural level, studies suggest that attention not only modulates activity of sensory neurons in various ways [15, 16], but contributes to ’hypothesis testing’ by making predictions about sensory information that should be encoded by lower neuronal levels [13, 17]. Behavioral studies also show that attention modulates perceptual and motor aspects of human behavior such as speed, reaction time, and performance accuracy [18]. A large number of attentional focus studies in the field of sport science and rehabilitation show that an external focus (i.e., focusing on the environmental task-relevant information outside of the performer’s body) enhances performance compared to an internal focus (i.e., focusing on the body or its movements) in a variety of motor tasks [19, 20]. For instance, an external focus improved performance accuracy and reduced pre-movement time relative to an internal focus during an isometric force production task [21]. These findings therefore may provide support for the role of attention in cognitive control of perception and its impact on motor outcomes. The constrained action hypothesis, as the most widely accepted theoretical explanation for external attentional focus effects, suggests that, unlike an internal focus that elicits controlled cognitive processing and impedes performance, an external focus enhances motor skill performance by invoking automatic processing that is characterized by faster and more reflexive adjustments [22].

Aside from attentional focus, mental imagery is another cognitive mechanism that may affect individuals’ perceptual-motor performance. Given the mental imagery definition as *“representations … of sensory information without a direct external stimulus”* [23] or *perceptual processing, in the absence of immediate sensory input from a relevant sense-modality* [24, 25], some researchers suggest that mental imagery has a critical role in cognitive control of perception [11]. Studies show that pre-cuing effects (e.g., increased visual search time) occur in the absence of any physical stimuli (i.e., it is not triggered by corresponding sensory stimulation) and it has been suggested that perception is indeed cognitively controlled by means of mental imagery rather than attention [11]. In other words, the argument is that individuals’ ability in creating mental images is the main modulator of perception. In the literature, these initial mental images have been considered as a type of attention known as preparatory attention (also known as attentional templet, attentional set, or search image) [26, 27]. Preparatory attention seems to be a phase in perception in which mental images of a given object are created in the sensory cortices (e.g., visual cortex) prior to presence of physical stimuli [27]. Furthermore, these initial mental images have identical, but reverse, perceptual processing path than sensory (e.g., visual) perception [28]. That is, while sensory perception involves a bottom-up neural network path from visual to frontal cortex, mental imagery involves a top-down direction from the frontal cortex to sensory areas (e.g., visual cortex). Therefore, attention and mental imagery, although mainly studied separately in sport science and rehabilitation, are closely inter-connected factors. To this extent, recent works suggest that mental imagery should be applied to attention [29] as both of them involve cognitive [30] and perceptual processes [28, 31] and share common neuro-cognitive circuits with perception [32, 33]. Furthermore, it seems that mental imagery is dependent upon attentional resources [34] and conversely, mental imagery may also impact attention [35]. Based on these findings, it seems quite reasonable to assume that mental imagery ability or individual differences in creating mental images modulates perceptual and motor aspects of performance during different attentional focus conditions. From a practical perspective, gaining more understanding in this regard would help practitioners to decide whether and how mental imagery should be applied to attentional focus strategies to further enhance perceptual and motor aspects of individuals’ performance.

A number of previous works have attempted to provide evidence regarding the modulating roles of *motor* imagery (MI: *a type of mental imagery* that is characterized by mentally imagining an action without any overt physical execution) [36] on motor performance and learning under different attentional focus conditions (i.e., internal versus external focus). In particular, MI modalities (i.e., kinesthetic MI: mentally ‘sensing’ proprioceptive or somatosensory aspects of movements, and visual MI: mentally ‘seeing’ different aspects related to performance such as distance and size) [37], and MI perspectives (i.e., internal visual imagery perspective: seeing from first-person perspective, and external visual imagery perspective: seeing from a third-person perspective) [37] during different attentional focus conditions have been investigated. Indeed, MI significantly modulates the effects of attentional focus during performance of different fine and gross motor tasks including trajectory tracing tasks, dart throwing, overhand ball throwing, and balance control [38–41]. Further investigations nevertheless are required for several reasons. Thus far, studies have yielded inconsistent results regarding the role of MI on motor performance and/or learning during different attentional focus conditions. In individuals with higher kinesthetic MI, an internal focus facilitated visuomotor performance (tracing a circular trajectory with a mouse-controlled cursor) and learning relative to an external focus, whereas individuals with higher visual MI benefitted from an external relative to an internal focus [38, 39, 42, 43]. Other studies however have failed to observe that kinesthetic MI facilitates performance and learning during any attentional focus condition [41, 44]. In addition, studies have mainly used relatively simple tasks including computer-based visuomotor tasks with a mouse (e.g., circle-tracing) [38, 42, 43] and dart throwing [44]. Therefore, there is a need to understand if MI modulates attentional focus effects in novices performing inherently more complex tasks (i.e., with relatively high index of difficulty). Furthermore, most of these works have investigated MI modalities (kinesthetic, vs. visual MI) without considering if MI *perspectives* (internal visual MI vs. external visual MI) have potential unique contributions to outcomes. To the best of our knowledge, only one recent study in children distinguished between MI perspectives (Bahmani et al. 2021), showing that while high levels of kinesthetic MI deteriorated overhand ball throwing learning in children adopting an external focus, external visual MI dominance resulted in superior motor learning for children adopting an external focus [41], suggesting that MI perspectives may also differently modulate attentional focus effects on motor performance. Finally, studies have focused on performance accuracy and there is little information about how attentional focus and MI interact to affect perceptual processing and movement time. In the present study, we sought to investigate whether MI modulates perceptual and motor functions under different attentional focus conditions. To determine whether perception is cognitively controlled by MI *or* attentional focus, and also given the existence of some inconsistencies regarding the role of kinesthetic MI on attentional focus effects, we employed additional tasks and outcome variables to complement traditional performance indicators related to end-point accuracy. To this end, we measured performance time, aiming point stability (i.e., aiming trace speed), and performance accuracy during shooting performance of a group of young novice 10-m air-pistol shooters.

### Method

#### Participants

Ninety-two young adult university students (M age = 21.84 ± 2.25 years; 29 females) with normal or corrected to normal vision, and with no self-reported musculoskeletal or postural disorders voluntarily participated in the study. Of note, a few incomplete datasets were excluded from the final analyses due to general optical system failure.^1^ All participants were novice (i.e., had no previous experience with the task) and naïve to the purpose of the study. The study was approved by Baghiatallah University of Medical Sciences review board (approval code: IR.BMSU.REC.1399.337).

### Imagery assessment

#### Movement Imagery Questionnaire-3 (MIQ-3)

The MIQ-3 is a 12-item questionnaire [45] to measure individuals’ ability (i.e., ease or difficulty) of generating mental imagery for four movements (knee lift, jumping, arm movement, and toe touch) via kinesthetic MI, internal visual MI, and external visual MI. The MIQ-3 asks participants to physically perform each movement first, then mentally imagine each movement. Participants were asked to rate each movement on a 7-point Likert scale from 1 (very hard to see/feel) to 7 (very easy to see/feel). Therefore, the maximum sum score that one could obtain in each subscale is 28.

### Apparatus and task

The participants were asked to shoot an air-pistol as accurately and as quickly as possible at an electronic target 10 m away in an indoor environment. The SCATT shooting system (SCATT Co., Russia) was used to quantify pistol shooting performance, congruent with prior work [46]. The SCATT system records the location of shots in two-dimensional space as a function of time throughout each shooting trial. This is accomplished using multiple optical camera devices, including a barrel-mounted light emitting and sensing unit with a reflective target border enabling the position of the aiming point to be recorded. The location of each shot was recorded as the position of the aiming point on the target at the time of the trigger pull, which was detected upon dry firing via a small microphone attached to the pistol. The system is comprised of a wired optical unit fixed on the pistol barrel and connected to a PC that automatically recorded all outcome variables. For the present study, we used accuracy, performance time, and aiming trace speed as our dependent/outcome (i.e., performance) variables of interest. Accuracy was determined by the position of the aiming point on the target at the time of trigger pull – two dimensional coordinates were converted to a ‘score’ via a series of concentric circles. A shot hitting the ‘bulls-eye’ of the target scored 10.9, the maximum possible score, with depreciating scores for each subsequent surrounding circle (the lowest possible score was ‘0’ if the shot missed the target entirely). Performance time was quantified as the time from lifting the gun to initiation of the trigger pull (in ms) and aiming trace speed was defined as the speed of pistol barrel (stability of hold of weapon stability) during the last second (mm/sec) [47, 48].

### Procedure

Following completion of the MIQ-3, participants began the shooting task. After a short familiarization period, participants were asked to shoot at the target in three different attentional focus conditions: (1) control condition, (2) IF, and (3) EF. Participants completed their shooting performance under control (no focus instruction) conditions first, then ordering of internal and EF conditions was counterbalanced between participants. Similar to previous attentional focus studies, we issued participants the control focus condition first, followed by the counterbalancing of attentional focus conditions, to ensure one condition could be used as a stable control that was not biased by previous attentional focus instruction (i.e., eliminate order effects for baseline performance, only) [49, 50]. While order effects may have reduced control task performance or improved internal or external focus performance (i.e., less ‘practice’ time given control task always performed first), the total number of trials for the entire experiment was relatively small, thus minimizing the potential for order effects in any condition [49]. Internal and EF instructions used in the current study were similar to previously published work on shooting performance that has demonstrated attentional focus effects [51]. In the IF condition participants were instructed to “focus on keeping your hand steady” and in the EF condition participants were instructed to “focus on keeping the gun steady.” In addition to attentional focus instructions, participants were also informed that they needed to complete each trial as accurately and as quickly as possible after seeing an optical signal. Participants completed 10 trials in each attentional focus condition, and participants’ scores in each condition were averaged to obtain total score of each condition.

### Data analysis

First, repeated measure analyses of variance were performed to investigate if shot accuracy was different among the attentional focus conditions. Similar analyses were done for performance time and aiming trace speed between attentional focus conditions. An alpha level of p < .05 was set a priori, and post-hoc analyses were performed using Bonferroni adjustments as appropriate. In addition, we ran simple regression analyses to investigate potential associations between each MI measure and each air-pistol shooting performance measure in different attentional focus conditions (i.e., between KMI and performance accuracy, between KMI and performance time, between KMI and aiming trace speed, between internal visual MI and performance accuracy, and so forth). Also, the association between performance time and shot accuracy, and between aiming trace speed and shot accuracy during each attentional focus condition were examined to help interpret findings. Finally, using MEMORE process macro [52], we ran separate simple model repeated measures analyses (see Montoya 2019) to investigate if each MIQ-3 score (i.e., global MI, internal visual MI, external visual MI, and kinesthetic MI) moderated performance during different attentional foci (i.e., during internal vs. control condition, external vs. control, and internal vs. external focus). Analyses were done for performance accuracy, performance time, and aiming trace speed that resulted in 12 separate simple model repeated measure analyses (4 MI measures: global MI, internal visual MI, external visual MI, and kinesthetic MI by 3 pair of attentional focus comparisons: internal focus vs. control, external focus vs. control, and internal focus vs. external focus). In addition to these primary analyses, we performed a secondary data analysis using motor imagery dominance to enhance reader interpretation (see Additional file 1). 

## Results

Descriptive statistics of participants’ internal visual MI, external visual MI, and kinesthetic MI scores are shown in Table 1.

**Table 1:** Descriptive statistics of participants’ internal visual MI, external visual MI, and kinesthetic MI scores

	Mean ± SD	Minimum	Maximum
Internal visual MI	5.81 ± 0.78	3.5	7
External visual MI	5.81 ± 0.0.75	4	7
Kinesthetic MI	5.87 ± 0.80	3	7
Total MI score	5.83 ± 0.69	4	7

### Repeated measures analysis of variance

**Fig. 1:**
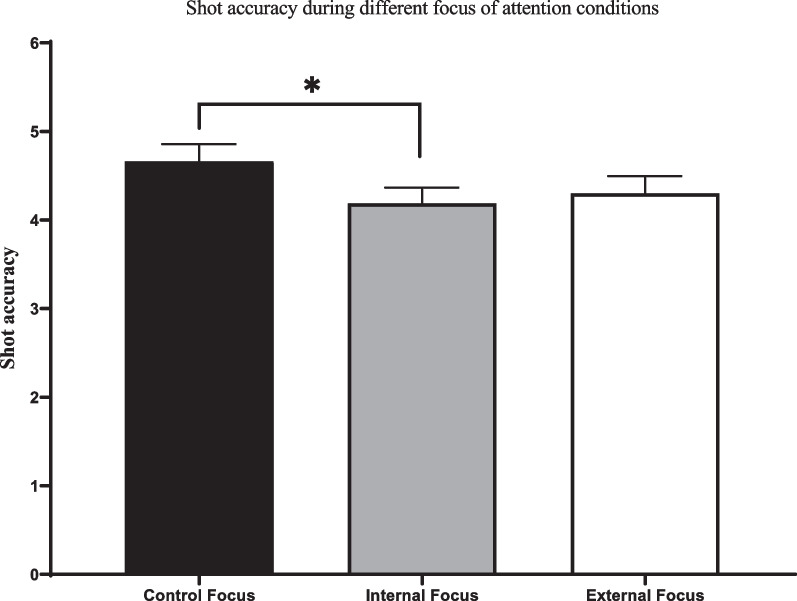
Mean and standard error (SE) of shot accuracy during different attentional focus conditions. As demonstrated by the * sign, participants had more accurate shots during control relative to the internal focus condition

**Fig. 2:**
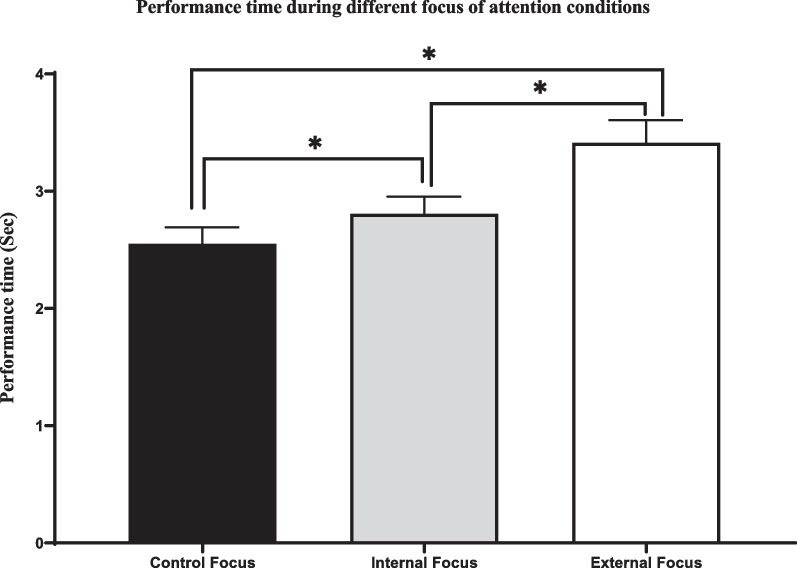
Mean and standard error (SE) of performance time during different attentional focus conditions. As demonstrated by the * sign, participants had longer performance time during external relative to the internal focus, and control condition. In addition, performance time was longer during internal focus relative to control condition

**Fig. 3:**
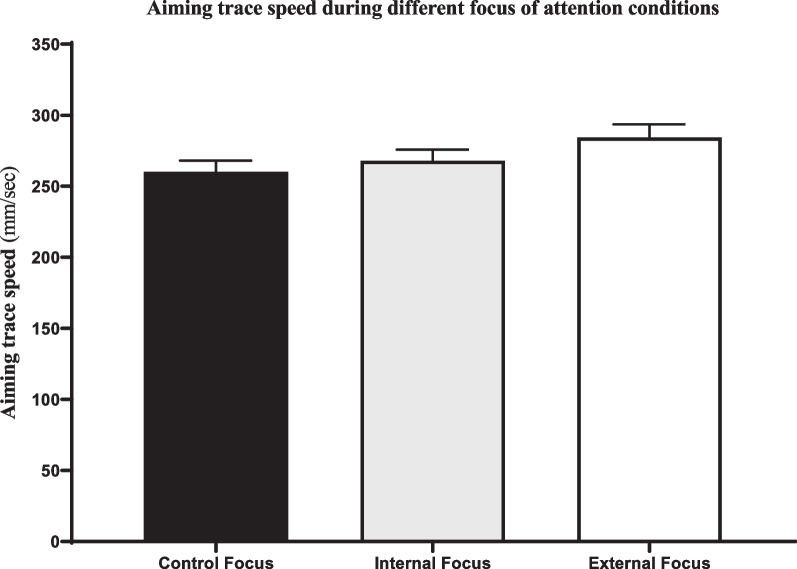
Mean and standard error (SE) of aiming trace speed during different attentional focus conditions. Although the effect of attentional focus conditions on aiming trace speed was significant, pairwise comparisons failed to identify the location of effect

Results indicated a significant difference in participants’ performance accuracy between attentional focus conditions (*F* (1, 85) = 4.45, *p* = .015, *η* = 0.046). Pairwise comparisons using Bonferroni adjustments revealed that while accuracy during external focus (*M* = 4.30, *SD* = 1.82) was not significantly different than other attentional focus conditions, participants were more accurate during control (*M* = 4.68, *SD* = 1.82) relative to internal focus (*M* = 4.19, *SD* = 1.68) condition (*p* = .017). Performance time was different between conditions (*F* (1, 81) = 21.47, *p* = .019, *η* = 0.21). Pairwise comparisons revealed that participants had higher performance time during external (*M* = 3.38 s, *SD* = 1.77) relative to both control (*M* = 2.46 s, *SD* = 1.15; *p* < .001) and internal focus (*M* = 2.78 s, *SD* = 1.27; *p* = .001), and performance time during internal focus was also significantly higher than during the control focus condition (*p* = .03). Finally, the effect of attentional focus conditions on aiming trace speed was significant (*F* (1, 81) = 3.38, *p* = .046, *η* = 0.041), although pairwise comparisons failed to identify the location of effect. Mean and standard error of shot accuracy, performance time, and aiming trace speed during different attentional focus conditions have been shown in Figs. 1, 2, and 3, respectively.

### Simple linear regression analyses

There were no associations between MI total score or its subscales with shot accuracy during any focus conditions (control: Total MI score *p* = .38, internal visual MI *p* = .35, external visual MI *p* = .81, kinesthetic MI *p* = .88; internal focus: Total MI score *p* = .97, internal visual MI *p* = .58, external visual MI *p* = .75, kinesthetic MI *p* = .71; external focus: Total MI score *p* = .76, internal visual MI *p* = .97, external visual MI *p* = .39, kinesthetic MI *p* = .54). There were no associations between MI total score or its subscales with performance time during any focus conditions (control: Total MI score *p* = .28, internal visual MI *p* = .56, external visual MI *p* = .94, kinesthetic MI *p* = .77; internal focus: Total MI score *p* = .26, internal visual MI *p* = .23, external visual MI *p* = .56, kinesthetic MI *p* = .32; external focus: Total MI score *p* = .17, internal visual MI *p* = .78, external visual MI *p* = .36, kinesthetic MI p = .94). There were no associations between MI total score or its MI subscales with aiming trace speed during any focus conditions (control: Total MI score *p* = .87, internal visual MI *p* = .88, external visual MI *p* = .07, kinesthetic MI *p* = 1.0; internal focus: Total MI score *p* = .60, internal visual MI *p* = .18, external visual MI *p* = .36, kinesthetic MI *p* = .79; external focus: Total MI score *p* = .69, internal visual MI *p* = .40, external visual MI *p* = .09, kinesthetic MI *p* = .33).

### Repeated measures moderation analyses

#### Shot accuracy

Neither total MI score (*p* range = 0.31 − 0.73) nor any of the three subscales (internal visual MI *p* range = 0.32 − 0.99; external visual MI *p* range = 0.34 − 0.84; kinesthetic MI *p* range = 0.10 − 0.37) moderated differences in shot accuracy between attentional focus condition.

#### Performance time

Neither total MI score (*p* range = 0.33 − 0.91) nor any of the three subscales (internal visual MI *p* range = 0.63 − 0.93; external visual MI *p* range = 0.58 − 0.84; kinesthetic MI *p* range = 0.07 − 0.62) moderated differences between foci of attention and performance time.

#### Aiming trace speed

Neither total MI score (*p* range = 0.69 − 0.70) nor any of the three subscales (internal visual MI *p* range = 0.36 − 0.79; external visual MI *p* range = 0.47 − 0.94; kinesthetic MI *p* range = 0.27 − 0.75) moderated differences between foci of attention and aiming trace speed.

## Discussion

In the present study, we investigated if MI and its subscales modulate cognitive control of perceptual and motor functions under different attentional focus conditions. To evaluate perceptual and motor outcomes, we measured movement time, shot accuracy, and movement stability (i.e., aiming trace speed) during different attentional focus conditions. Based on several lines of research, we expected that individual differences in MI would play a crucial modulating role in cognitive control of both perceptual and motor performance. Our findings however failed to show any modulating role for MI. We nevertheless observed that attentional focus affected perceptual and motor outcomes measured in this study. More particularly, an internal focus resulted in less accurate shots than control focus. In addition, movement time was shorter during control relative to both internal and external focus, whereas an external focus resulted in longer movement times than other attentional foci.

Several attentional focus hypotheses, including the constrained-action hypothesis and self-invoking trigger hypothesis, have previously suggested that an internal focus hinders perceptual and motor functions due to controlled processing that interferes with automatic performance [22, 53, 54]. Instead, it has been suggested that an external focus improves performance relative to an internal focus by invoking more automatic and more reflexive (faster) adjustments [22]. Our findings partially (but not fully) supported these hypotheses as the internal focus was associated with less accurate shots with longer movement times only compared to control focus condition. Indeed, while shot accuracy during the external focus was not different than the internal focus, the external focus was associated with longer movement times (i.e., extended cognitive processing) than the internal and control focus – a finding that seems contrary with previous reports and the constrained-action hypothesis [19]. While it is difficult to explain why an external focus resulted in longer movements compared to other conditions, it may be attributed to task difficulty and skill level. Although the present task was a standard shooting task, it may be considered *too* difficult for our inexperienced individuals as they had to shoot towards a standard, yet relatively small bulls-eye (11.5 mm diameter) from a distance of 10 m. Prior literature proposed a hypothesis that suggests *overly* difficult tasks (i.e., tasks with high index of difficulty) do not allow for beneficial adoption of superior cognitive strategies such as an external attentional focus [55], possibly due to imposing information overload from doing difficult tasks [56]. Although Yamada et al. (2022) failed to fully support their hypothesis, other studies also suggest that task difficulty could be a potential modulator of attentional focus effects on motor behavior, with low or medium difficulty tasks ideal for achieving beneficial performance and learning outcomes when adopting an external attentional focus strategy [57–60]. Our study, along with previous findings, highlight the importance of considering task difficulty in future investigations exploring the role of attentional focus on motor performance.

Given an external focus did not facilitate our participants’ accuracy while increasing their performance time, alternative explanations are warranted given the extant literature on this topic (external focus broadly superior for motor performance; Wulf 2013). For instance, during control processes of goal-directed aiming tasks, successful visual processing is necessary to achieve desired movement accuracy [61]. Future studies may want to quantify visual processing ability and performance (e.g., eye tracking) to isolate its relative influence on the relationship between movement time and performance accuracy under different attentional focus conditions. In addition, increased movement time during both internal and external attentional focus conditions relative to control focus condition may be attributed to increased perceptual processing (cognitive processing) needed to process additional attentional verbal cues (either internal or external focus cues). Prior studies show that the number of attentional verbal cues may increase cognitive workload [51] and hinder skilled motor performance [62].

Our study failed to provide support for the suggestion that perception is cognitively controlled by means of mental imagery [11]. Therefore our findings contradict the suggestion that MI should be applied to attentional focus strategies [29]. Our findings are also in contrast to several recent works which investigated the role of MI on motor behavior during different attentional focus conditions. Prior works mainly suggested that performance is greater when visual MI dominant individuals perform a task under an external focus [38, 41, 44], whereas individuals with high kinesthetic MI dominance benefit from adopting an internal relative to an external focus strategy [38, 39, 42, 43]. In this particular, it has been suggested that adopting an optimal (congruent) attentional strategy (i.e., an internal focus for individuals with higher kinesthetic MI dominance and an external focus for individuals with higher visual MI dominance) may enhance neural efficiency by reducing neural activity of parietal and frontal brain regions [43]. We consider our findings important as our data indicates that attentional strategies affect perceptual and motor outcomes independent of novice 10-m air-pistol shooters’ baseline MI abilities, ultimately suggesting a unique contribution for attention in cognitive control of perception and action. Nevertheless, the lack of moderating effects could be due to several reasons. *First*, despite including a relatively large number of participants in the study, our participants were not particularly poor at motor imagery, as our participants had self-reported MI scores that were relatively higher than a score that can be deemed as poor imagery ability. In the MIQ-3, a score of 4 is neutral (neither easy nor hard), and individuals that score 3 or lower are those who report difficulty in MI ability. As our descriptive results show, our participants had mean MI scores (including MI modalities and perspectives) that are considered relatively high (range from 5.81 to 5.87). However, rather than considering this as a methodological limitation of the present work, we consider this a unique finding as individuals in our study had less difficulty imagining their action from different modalities and perspectives, compared with previous works reporting relatively high MI abilities [45, 63–65]. For example, McNeill et al. [64] conducted a study to investigate if good kinesthetic MI participants [64] exhibit greater performance improvements than poor kinesthetic MI participants following an MI intervention and failed to reveal between-group differences in mean golf putting accuracy. The lack of differences has been attributed to the classification method as McNeill et al. [64] considered the kinesthetic MI median scores less than 6 as ‘poor’ kinesthetic imagers, while the score 6 is a relatively high score on the 7-point MIQ-3 Likert-type scale [66]. Therefore, our study suggests that MI differences in typical individuals cannot modulate performance, probably because individual differences in MI ability were not of sufficient magnitude or variability. Another possible explanation for our current findings may be that individuals with high MI scores choose attentional focus strategies independently from their MI traits. That is, since our participants had relatively high MI scores in all MI subscales, their relatively high ‘overall’ MI abilities allowed for greater flexibility to choose attentional focus strategies. In other words, while MI moderation effects could result from lacking certain MI ability, high overall MI abilities may allow individuals to use attention in a more flexible manner. *Second*, although, like many previously published pistol-shooting-related work [51, 67, 68], our measure of shot accuracy may not have been granular enough to detect subtle and small changes in motor performance (see Fischman, 2015). Adding complementary measures, such as ‘performance consistency’ may better detect performance outcomes using attentional focus and MI manipulations. *Third*, We should also note that our participants were novices, and therefore their little experience of physically executing the imagined action may have resulted in inefficient (unorganized) neurocognitive processing [69], that as a consequence, may have hindered the role of MI on motor performance. Another possibility because attentional foci and MI failed to moderate performance accuracy may be due to excessive task difficulty. As our results demonstrate, mean shooting accuracy was relatively low whereas standard deviation of shooting accuracy was relatively large. Novices’ performance was understandably poor and highly variable, and thus potentially less modifiable by attentional focus and/or MI. Future studies may want to replicate this work by adding a group of skilled or expert individuals to their study. *Fourth*, the MIQ-3 used in our current study does not measure some important aspects of mental imagery such as mental imagery vividness- a diagnosis tool for identifying individuals with extreme imagery conditions [70–72] which affects other cognitive functions such as memory and attention [73–77]. Furthermore, more than one MI ability may interact to moderate attentional focus effects on motor performance. Therefore, future work should consider different MI abilities additively and uniquely influence cognitive control of perception under different attentional focus strategies. *Fifth*, and finally, while MI may be action specific [78], the MIQ-3 evaluates individuals’ ability to mentally imagine very basic actions that may not predict individuals’ ability or tendency in shooting.

Our study has some limitations that should be acknowledged. We used a laboratory-based task that may reduce the complexity of what occurs during real situations [79]; therefore, other studies should investigate how MI and attentional focus affect performance in real, highly demanding situations. In addition, although we adapted a previously published approach to direct participants’ attention during an internal focus, we acknowledge that it may not be the most precise method. In particular, we asked participants to “focus on keeping your hand steady.” Although this instruction is an internal focus instruction, it may cause visual dominant imagers to focus visually on their hand to see their hand mentally rather than ‘feel’ it. Future studies should consider additional instructional manipulations, such as directing individuals’ focus to more sensory-related factors like pressure, or force produced by body during a particular motor task to extend the current work. Additionally, although we kept the number of trials in each condition relatively small to minimize the potential of order effects (see the methods section for more details), it is not clear whether this methods decision ultimately influenced any of the performance outcomes. Future work may want to replicate this study by fully counterbalancing all three study groups. We also did not use manipulation checks or interviews to measure participants’ adherence to our instructions. While lack of manipulation checks could potentially limit our findings, previously published attentional focus studies have shown high levels of adherence to similar attentional focus instructions [41, 80–86]. Additionally, several individual factors such as stress, arousal and anxiety were not controlled in this study which warrants caution in broad interpretation of our findings. Finally, relatively few number of females (about 31% of participants) volunteered to participate in this study, thus limiting the generalizability of the study findings to males.

## Conclusion

In conclusion, this study showed that participants’ self-reported baseline MI and its subscales do not moderate attentional focus effects on novices’ air-pistol shooting performance accuracy, performance time, and aiming trace speeds. These findings may suggest that individuals’ MI does not affect cognitive control of perceptual and motor functions. However, an internal focus may reduce shot accuracy relative to control focus. Importantly, an external focus may result in an increased performance time to ensure the task goal. The study highlights the importance of future studies to enhance our theoretical and practical knowledge regarding mental imagery, and attentional focus in sport science and rehabilitation.

## Supplementary Information

**Additiona file 1.** **Supplementary materials.** Secondary data analysis using motor imagery dominance.

## Data Availability

The datasets used and/or analysed during the current study are available from the corresponding author if requested.

## Abbreviations

MI: Motor imagery
MIQ-3: Movement imagery questionnaire-3.

## Footnotes

1 While 92 participants completed the study, accuracy, performance time, and aiming trace speed data of some participants were incomplete for technical reasons. Specifically, shooting accuracy data from 6 participants (2 males, and 4 females) was not provided by the SCATT system (optical cameras failed to track and record aiming point, precluding system/CPU calculation of accuracy relative to concentric circles). Likewise, the SCATT system failed to provide performance time and aiming trace speed data from 4 additional participants (3 males, and 1 female) for the same data acquisition technical limitations (general optical camera system failure). Consequently, the total sample size reduced to 86, 82, and 82 for accuracy, performance time, and aiming trace speed, respectively.
