# Supplementary material for: Is cognitive control of perception and action via attentional focus moderated by motor imagery?
Source: BMC Psychol. 2023 Jan 16;11:12. doi: 10.1186/s40359-023-01047-z (PMC9841651; doi:10.1186/s40359-023-01047-z)
Supplement: Supplementary file 1 — Additiona file 1. Supplementary materials. Secondary data analysis using motor imagery dominance. [file 40359_2023_1047_MOESM1_ESM.docx]

Supplementary materials

**Secondary data analysis using motor imagery dominance:**

In previous studies supporting the MI moderation effect, researchers usually categorized participants into groups according to the questionnaire scores of different MI types and found interaction between MI groups and attention focus conditions, which could not exclude other between factors of groups. Therefore, we performed a secondary data analysis commensurate with previous literature to enhance reader interpretation and support greater transparency in the study findings. For the secondary data analysis, we first categorized our participants to either kinesthetic or visual dominant groups. Congruent with prior work (Sakurada, Hirai, & Watanabe, 2019), kinesthetic dominant participants were defined as individuals with *relatively* higher kinesthetic than visual imagery scores (i.e., kinesthetic MI score minus visual imagery score > 0). Visual dominant participants were defined as participants with *relatively* lower kinesthetic than visual imagery scores (i.e., kinesthetic MI score minus visual imagery score < 0). An independent samples *t*-test was conducted to evaluate whether there were significant between-group differences in visual and kinesthetic scores for the two groups. Then separate 2 (MI group: kinesthetic vs visual) * 3 (attentional focus conditions) repeated measure analysis of variance was conducted for each dependent variable.

Imagery scores

Results indicated that kinesthetic dominant group had higher kinesthetic imagery scores (*M =6.10*, *SD* = .68*)* than visual dominant group (*M = 5.47, SD = .*80, *t* (77) = 3.77, *p* < .001). Our visual dominant participants also had higher external visual imagery scores (*M = 5.99, SD =* .69*)* than kinesthetic dominant participants (*M = 5.62*, *SD* = .70, *t* (77) = 2.34, *p* = .022). Kinesthetic (*M = 5.72, SD =* .85*)* and visual dominant participants (*M = 5.81, SD =* .62*)*, however, were not significantly different in internal visual imagery scores, *t* (77) = .50, *p* = .62.


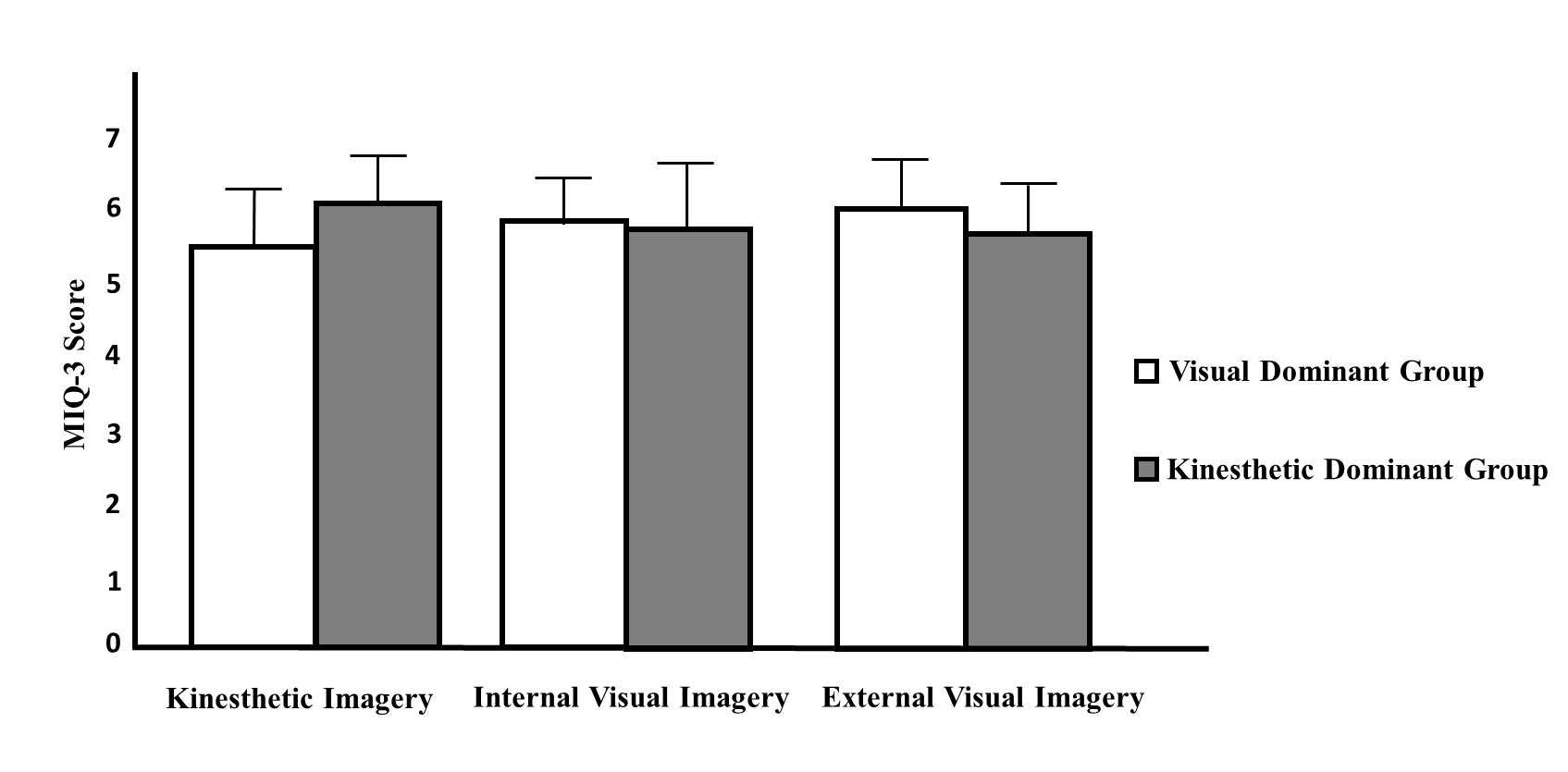


*Shot accuracy*

Results indicated that the main effect of attentional focus was significant *F* (1, 73) = 3.85, *p* =.023, *η* = .05). Post hoc analyses using Bonferroni adjustments revealed that participants had more accurate shots during control (*M* = 4.66, *SD* = 1.83) than internal focus (*M=* 4.14, *SD* = 1.67) condition. The main effect of MI dominance and MI dominance by attentional focus interaction were however non-significant (*P_s_* > .05).


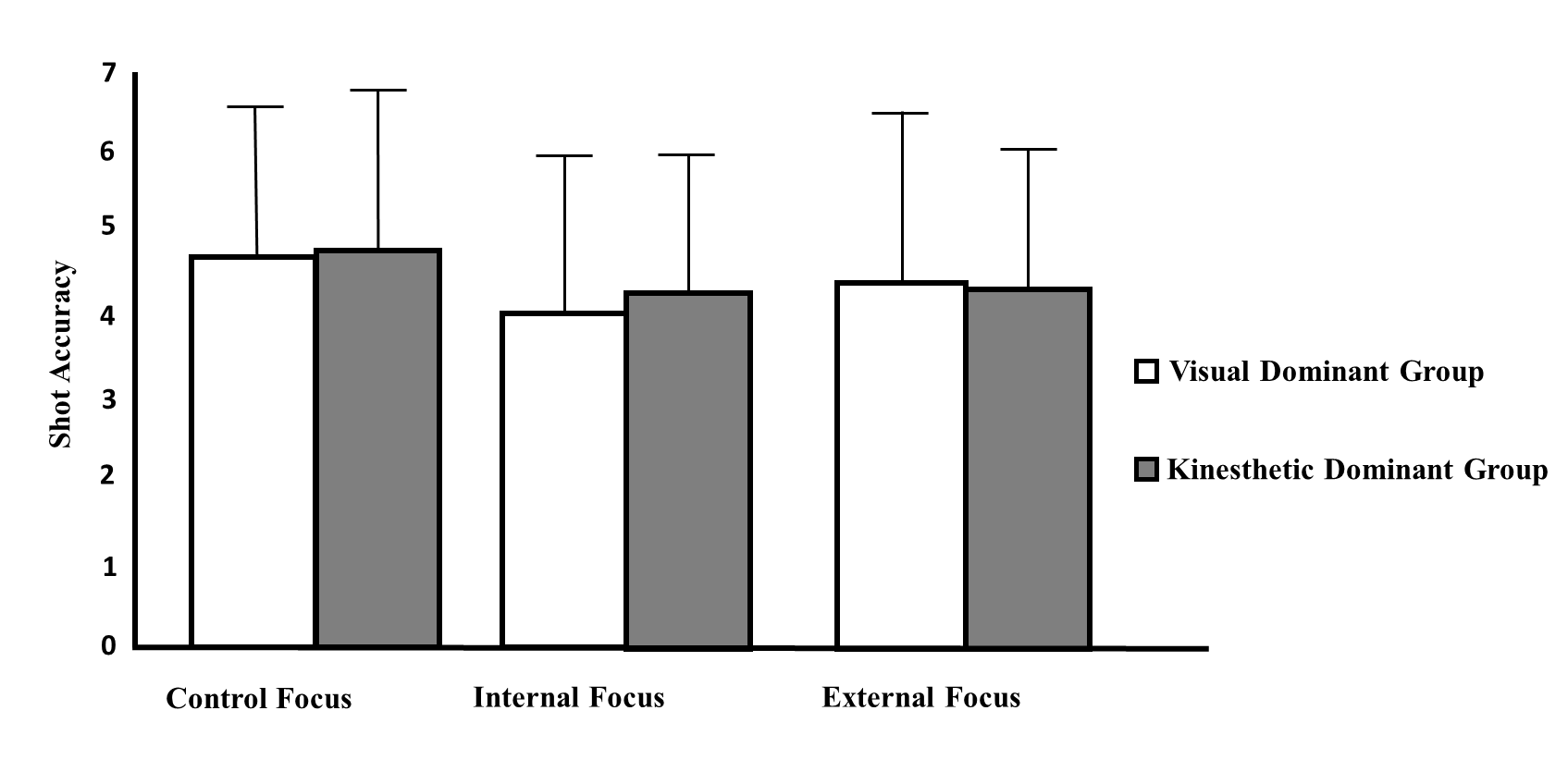


*Performance time*

The main effect of attentional focus was significant *F* (1, 73) = 16.79, *p* < .001, *η* = .19) with longer performance time during the external (*M* = 3.33, *SD* = 1.65) relative to both internal (*M* = 2.74, *SD* = 1.24) and control focus (*M* = 2.47, *SD* = 1.17) conditions. The main effect of MI, and MI * attentional focus were however not significant (*P_s_* > .05).


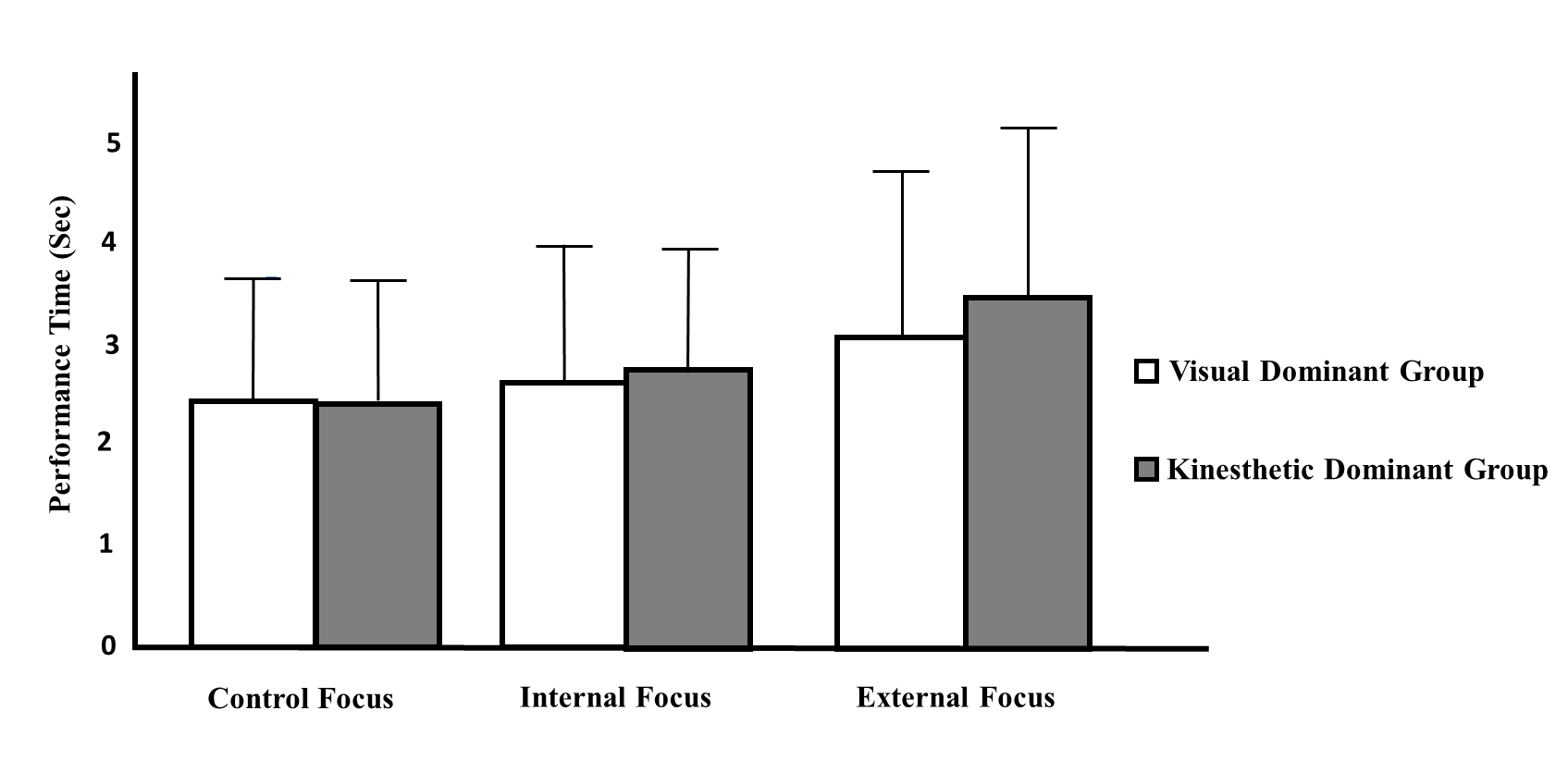


*Aiming trace speed*

The main effect attentional focus and MI * attentional focus were not significant (*P_s_* > .05). However, a marginal main effect was found for MI *F* (1, 73) = 16.79, *p =*.057, *η* = .19) with higher aiming trace speed for visual (*M* = 281.04, *SD* = 71.26) than kinesthetic (*M* = 253.67, *SD* = 66.91) MI dominant participants.


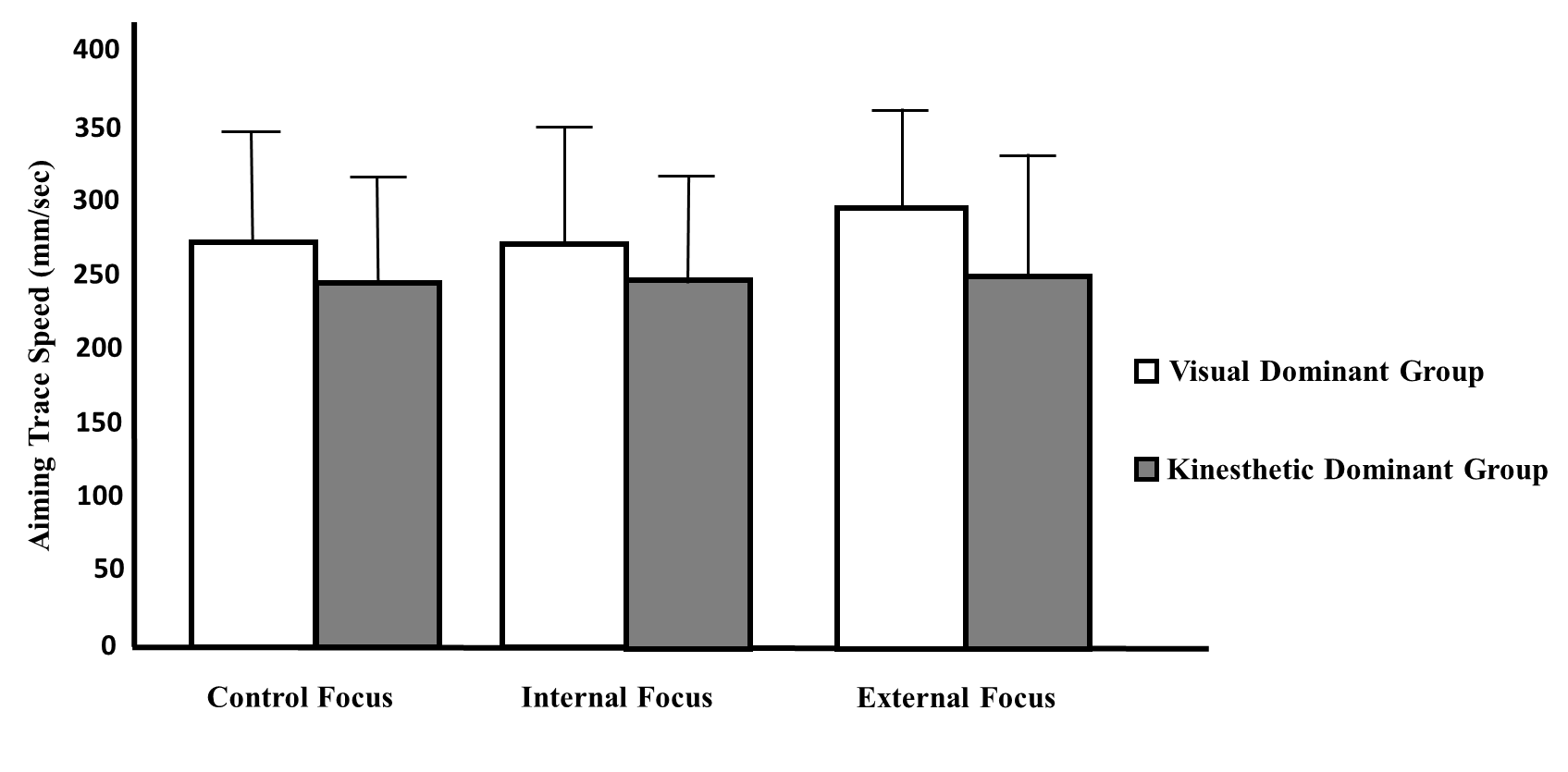


Sakurada, T., Hirai, M., & Watanabe, E. (2019). Individual optimal attentional strategy during implicit motor learning boosts frontoparietal neural processing efficiency: A functional near‐infrared spectroscopy study. *Brain and behavior, 9*(1), e01183.
